# Collective Philanthropy: Describing and Modeling the Ecology of Giving

**DOI:** 10.1371/journal.pone.0098876

**Published:** 2014-07-01

**Authors:** William L. Gottesman, Andrew James Reagan, Peter Sheridan Dodds

**Affiliations:** 1 Department of Mathematics and Statistics, University of Vermont, Burlington, Vermont, United States of America; 2 Center for Complex Systems, University of Vermont, Burlington, Vermont, United States of America; 3 Computational Story Lab, University of Vermont, Burlington, Vermont, United States of America; 4 Vermont Advanced Computing Core, University of Vermont, Burlington, Vermont, United States of America; Max Planck Institute for the Physics of Complex Systems, Germany

## Abstract

Reflective of income and wealth distributions, philanthropic gifting appears to follow an approximate power-law size distribution as measured by the size of gifts received by individual institutions. We explore the ecology of gifting by analysing data sets of individual gifts for a diverse group of institutions dedicated to education, medicine, art, public support, and religion. We find that the detailed forms of gift-size distributions differ across but are relatively constant within charity categories. We construct a model for how a donor's income affects their giving preferences in different charity categories, offering a mechanistic explanation for variations in institutional gift-size distributions. We discuss how knowledge of gift-sized distributions may be used to assess an institution's gift-giving profile, to help set fundraising goals, and to design an institution-specific giving pyramid.

## Introduction

The scope and health of philanthropic institutions contribute substantively to the cultural and economic well-being of a great diversity of societal institutions. Between 1970 and 2010 Americans gave approximately 2% of their disposable income to philanthropic causes [1]. The distribution of income in the United States and many other countries has long been described by various heavy tailed distributions including power law, log-normal, Boltzman, and combinations thereof [2]–[4]. Similar distributions have been found in the size of gifts to charitable causes [5].

Here, our aims are to (1) examine empirical data for an approximate power-law size distribution model of philanthropic behavior; (2) describe a general mathematical model for philanthropic gifting in a manner that gives greater insight into how different organizations raise money and how individuals choose the amounts of their gifts; and (3) explore the usefulness of our findings on current fundraising practices [6]. We have chosen the power law distributions for the sake of simplicity and to aid development of a primitive model describing heavy-tailed gifting behavior capable of addressing basic questions about philanthropy. We wish to emphasize that we do not claim that gift size distributions are perfectly described as ‘true’ power laws generated by some underlying mechanism(s) not yet elucidated. Rather, we use power law approximations—linear approximations in logarithmic coordinates—to gain some traction in our description and to provide a way to carry out some idealized analysis, fully appreciating the appromixate nature of our work. Larger, much more comprehensive data sets will certainly advance our understanding beyond what we have been able to achieve here.

As a foundation for our investigations, we have constructed a data set spanning a wide range of institutional categories. We obtained anonymous gift data for a total of six institutions:

- two educational institutions: University of Vermont, Burlington, VT, and Albert Einstein Medical School, Bronx, NY,
- one health care institution: Mt. Sinai Hospital in Manhattan, NY,
- one combined purpose organization: United Way of Chittenden County, VT,
- one local cultural and educational organization: ECHO Science Center in Burlington, VT,
- and one arts center: Flynn Theater in Burlington, VT.

We provide the complete data set as part of Dataset S1.

Our central characterization of gift-size distributions will be through measuring power-law exponents for the paired statistics of Zipf distributions [7] and gift-size frequency distributions, and we explain both now. First, the Zipf distribution for a list of gifts is generated by ranking gifts in order of descending monetary size. Writing gift size as 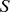 and gift rank as 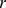, an ideal Zipf distribution obeys:
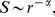
(1)where we will call 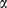 the Zipf exponent. Alternately, we can compile a gift-size frequency distribution: for each gift size 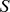, we record the number of such gifts 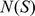. Again for an ideal system, we would observe
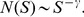
(2)Both views have their merits: Zipf distributions follow a very natural construction and are simple to interpret, while power-law size distributions most clearly represent a system's probabilistic behavior. Later in our analyses, we will consider the probability density 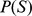, the normalized version of 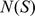.

We can show that the two distributions are related by considering the complementary cumulative distribution function, the number of gifts of at least size 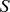: 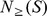. We see that 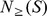 is equivalent to the rank of 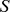, meaning 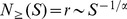 using Eq. (1). A simple calculation starting from the size frequency distribution gives 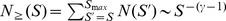. The exponents are therefore connected as:
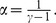
(3)Empirically, a typical range for 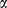 is 1/2 to 1 with these limits corresponding to 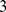 and 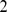 for 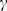. For 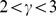, we have the ‘statistics of surprise’: gifts are typically small but the variance is very large being dominated by the largest gifts. If 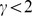, we have an even more extreme circumstance of the average gift size being typically large as well.

In what follows, we will generally present figures showing Zipf's distribution. We will estimate Zipf's 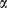 with a Maximum Likelihood (ML) approach [8], [9], and then determine 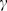 using Eq. (3). We provide details of these calculations including comparisons to other potential distributions in the Methods section and in Fig. S1 and Tabs. S1 and S2 in File S1). In spite of our choice for figures and for the purposes of analysis, we will prefer to describe our findings using the gift-size distribution exponent 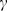, though occasionally we will use Zipf's 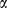 when more convenient.

Due to the real-world nature of our data sets, our measurements are necessarily not exact. Further, we are not stating that all philanthropic gift-size distributions possess idealized power-law tails. Power-law statistics are notoriously difficult to estimate and, moreover, convincingly showing that a power law even applies is itself a fraught endeavor [8], [10]. Nevertheless, assuming approximate power-law tails is reasonable and gives us a serviceable diagnostic tool for building and challenging our descriptions and theory.

We report on our work as follows. We first present and give an overall analysis of our six philanthropic-giving data sets. We then propose an explanation for the variation in gift-size distributions across institutions, based on the gift-giving preferences of individual donors. Based on our findings, we then give recommendations for fundraisers concerning the so-called ‘top 12 rule’, fundraising pyramids, organization fundraising capacity, and data collection.

## Results

**Figure 1:**
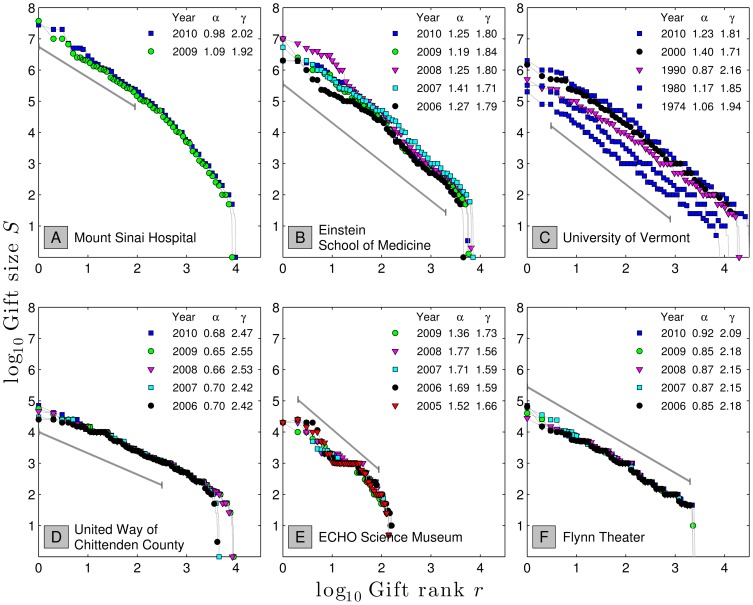
Gift size distributions for a range of institutions. The reported 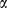 and 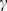 were fitted to the region indicated by solid grey line, and the 95% CI of this fit, as well as year for which the fit is plotted, are included for each organization. The ranges over which the data were fit was chosen empirically; other approaches were found to be inconsistent (see Methods). **A.** Health Care: Mt. Sinai Hospital, 2010 had 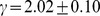. **B.** Higher Education (Medical): Einstein School of Medicine, 2010 had 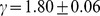. **C.** Higher Education (General): University of Vermont, 2010 had 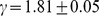. **D.** Combined Purpose: United Way, 2010 had 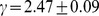. **E.** Cultural: ECHO Aquarium and Science Center, 2009 had 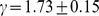. **F.** Performing Arts: Flynn Theater, Burlington VT, 2010 had 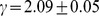. Later in Fig. 11, we show similar data for an anonymous religious institution. Dates and amounts of all contributions were collected over time periods ranging from 2 years to 37 years. United Way and Mt. Sinai Hospital were the only organizations able to ensure that annual donor gift amounts reflected that year's total of donations by a single donor, rather than individually posting multiple gifts made by a single donor during that year.

In Fig. 1, we show gift-size Zipf distributions for our six organizations, organized by calendar year for a total of 27 distributions. For each year's distribution for each institution, we estimate 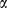 and 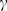, indicating the fitting region with a solid grey line.

Our initial observation is that data for each organization in Fig. 1 is highly skewed and are generally well fit by decaying power laws. Four of the six institutions are particularly robust with the exceptions being Mt. Sinai Hospital (Fig. 1A), which deviates from a simple power law after the first few hundred donors, and ECHO Science Museum, which shows the effect of providing strong gift categories, leading in its case to a shelf at $1000 (Fig. 1E). Examples of similar smaller shelves can be seen in the other distributions at natural values of $50, $100, and so on.

We also see that institutions show remarkable consistency across years. For example, in the case of the University of Vermont (Fig. 1C), we see Zipf's 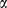 and its related 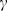 are relatively stable across three decades of gift rank, as well as over a range of 8,000 to 31,000 gifts per year. We also see that idiosyncratic distributions such as those of Mount Sinai (break in scaling, Fig. 1A) and ECHO Science Museum ($1000 shelf, Fig. 1E) are strongly preserved from year to year.

As mentioned above, smaller values of 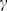 are associated with more extreme distributions skewed towards very large gifts. The two educational institutions possess extreme distributions with 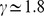
–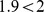, and their average gift sizes are relatively large. By contrast, United Way has a 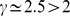 meaning its average gift is small but large ones are possible.

To provide an initial summary, these distributions suggest four notable characteristics of philanthropic gifting:

1. The distribution of the size of philanthropic gifts received is qualitatively described with a power-law relationship.
2. Within a given institution, the gift-size distribution exponent 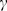 remains nearly constant year-to-year.
3. As indicated by the similar values of 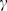 for the two higher education institutions, 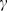 may be relatively constant within a single philanthropic category.
4. The gift-size distribution exponent 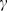 varies considerably between philanthropic categories.

Of the number of questions raised by these observations, we will focus in particular on one: Why does 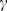 vary among different categories of philanthropic institutions? Our concrete goal will be to model how giving behavior of people at different income levels influences the gift-size exponent 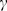.

**Figure 2:**
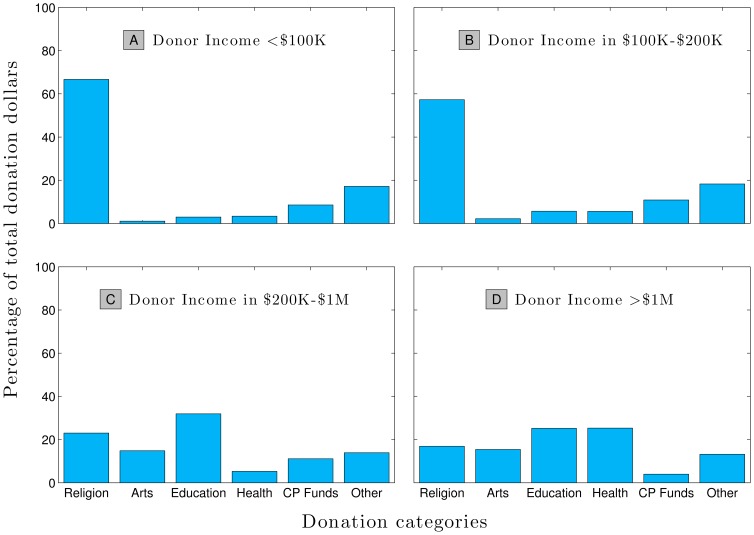
Data showing how donors of different income groups distribute their charitable giving (United States, 2005) [11]. For example, on average donors earning less than $100,000 chose to direct 67% of their total giving to religious causes, panel **A**, but donors earning more than one million dollars chose instead to direct 17%, panel **D**. CP Funds stands for Combined Purpose Funds.

As we show in Fig. 2, there is considerable variation in donor behavior based on income, and this provides some insight for our next step forward. The data we use here comes from the Indiana University Center for Philanthropy 2005 Study on Charitable Giving by Income Group which found in particular that a person's income level is strongly informative of the type of institution they prefer to support [11]. Donors earning less than $100,000 per year, for example, give a higher percent of their philanthropic dollars (8.6%) to combined purpose funds (e.g. United Way) than do those earning more than $1,000,000 per year and direct only 4% of their philanthropic dollars toward such charities. The opposite is true for education, toward which donors with incomes less than $100,000 direct only 3% of their philanthropic dollars, while people earning more than $1,000,000 direct 25% of their giving. As such, we would expect that educational institutions would have a much lower 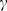 (associated with higher average gift sizes) than combined purpose funds. Our data bears this out, with a 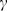 of 1.81 for University of Vermont, and 2.47 for United Way of Chittenden County (2010).

**Figure 3:**
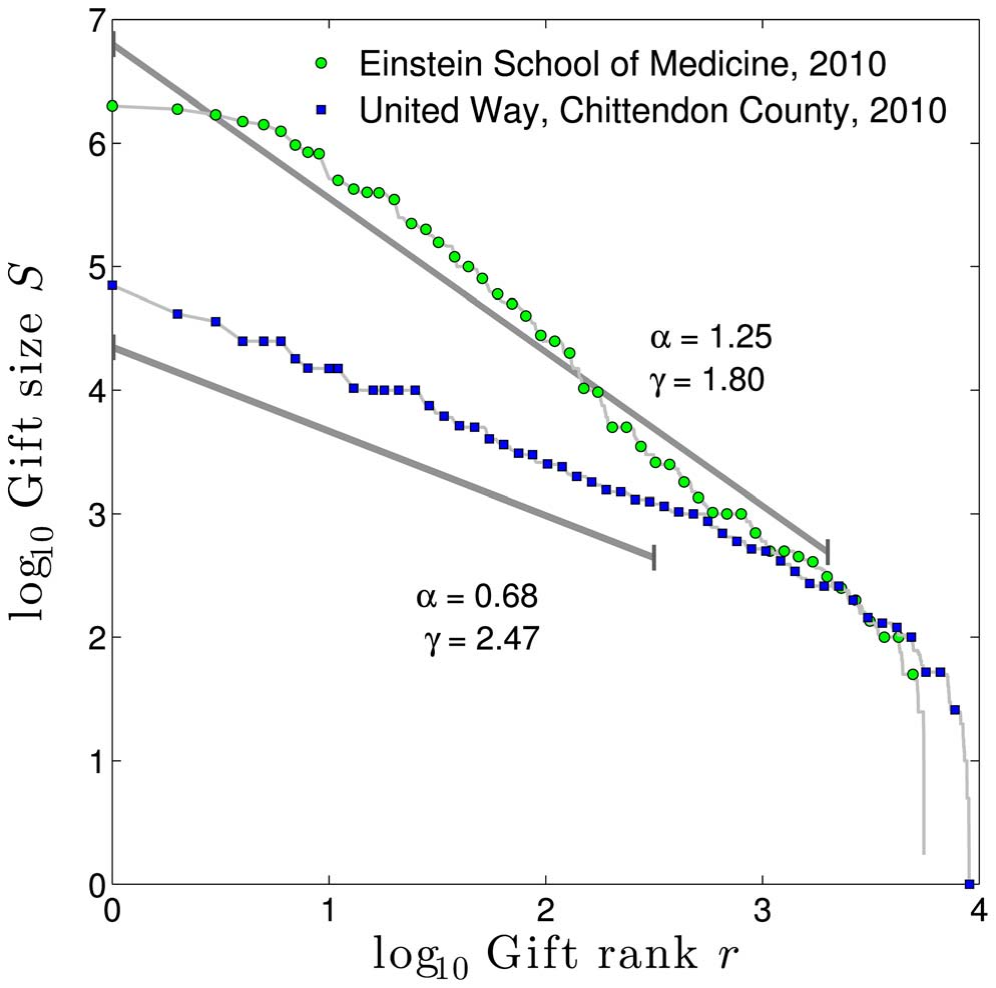
Comparison of 2010 giving to two organizations with a similar number of donors, and similarly sized smaller gifts. Despite these similarities, The Albert Einstein School of Medicine was able to attract top ranked gifts that were approximately 30 times larger than those of the United Way of Chittenden County, and raise 10 times the total, because Einstein enjoyed a substantially lower 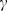 than United Way.

To make some headway with this issue of varying 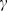, we first need to examine how gift-size distributions differ in more detail. In Fig. 3, we show that the Albert Einstein School of Medicine and the United Way of Chittenden County have a similar sized donor base, both with the 2000th donor giving approximately $200, yet the largest gifts to Einstein are roughly 30 times those of the United Way. How do we explain this? The answer is not as simple as that all donors to Einstein give a multiple of what they would give the the United Way: this would not change the slope (i.e., 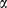 or 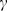) of the Zipf or frequency distributions in log-log space.

As a first attempt, we start with the reasonable assumption that larger donations originate from wealthier donors. In terms of Zipf distributions, gift sizes will be ranked in the same order as the donors who give them, according to their wealth. If we therefore know, for a given time period, the distribution of the total amount donated by each individual across a population, we can estimate how much individuals, as a function of their income, must relatively give to specific charity categories to obtain the specific distributions (i.e., values of 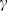) we observe in Fig. 1.

**Figure 4:**
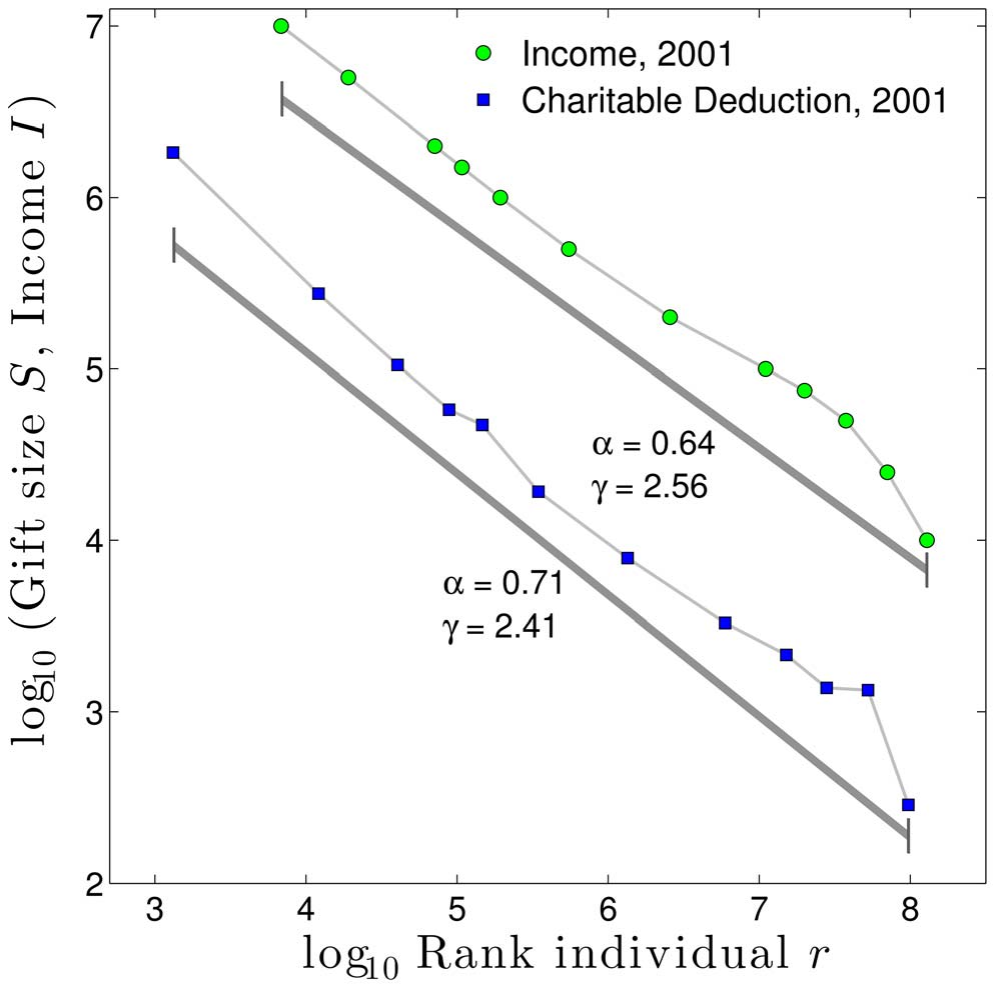
Data from the United States Internal Revenue Service (IRS) 2001 tax returns for personal income and charitable deductions. On average, people claimed charitable deductions at a rate of 2.9% of their income. The top 0.15% of tax filers gave at a higher rate averaging 4.8%, resulting in a 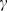 for charitable deductions slightly lower (2.41) that that for income (2.56). Fits were computed using linear regression in log-log space, after attempts to use maximum likelihood methods failed due to finite size bias. Since income was reported as bin average, we found the rank of the individual with that average by assuming a power law distribution within each bin with 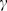 equal to the fit for the whole distribution. This procedure was bootstrapped (as the new individual ranks changes the whole distribution 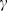) until convergence of 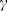 within 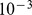.

With this motivation, we turn to evidence that describes how income and gift-giving are related within the United States. The United States Internal Revenue Service (IRS) 2001 tax return data shown in Fig. 4 compares reported income to reported charitable deductions. On average, Americans donated 2.9% of their income, with deductions for charitable giving appearing nearly proportionate to income. When plotted in Zipf format and fitted to a power law distribution, 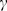 for charitable giving (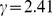) is slightly smaller than that for income (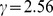), favoring donating a slightly higher percentage of income by wealthier individuals.

To move ahead, we now need to be able to compare two arbitrary Zipf distributions whether they be Zipf distributions of individual wealth or gift sizes. For ease of language, consider gifts given to a specific institution with 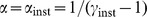, and total donations made by individuals in a population with 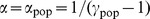. We want to know how the first ranked (largest) donation to the institution compares with the first total amount donated by the population, and so on, down to the last ranked donation. We derive this relationship by starting with the Zipf distributions:
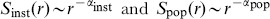
(4)which, by isolating and equating ranks, immediately gives us
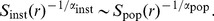
(5)Using 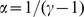, we then have that the size 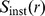 of a gift to the institution is related to the similarly ranked total amount donated by an individual 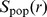 according to
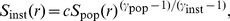
(6)where 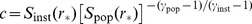 with 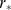 being any reference ranking.

We determine how much two individuals 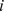 and 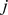 in our theoretical population relatively give to the institution. If these individuals have wealth ranks 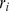 and 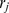 (which by assumption are their donation ranks as well), then using Eq. (6), we have
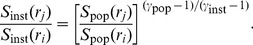
(7)Finally, we can compute a multiplier 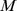 which is the ratio of gift sizes to an institution normalized by the total donated by individuals 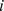 and 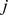:
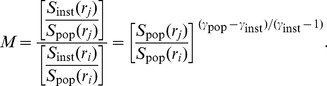
(8)


We can now use Eq. (8) to transform the distribution of personal giving (and relatedly, that of income) into the distributions for giving to various categories of philanthropy. First, we estimate 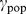 using the 2001 IRS charitable deduction data as a reference distribution, giving 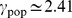. Employing Eq. (8), we then calculate multipliers for what people of different total donating levels would have to give to achieve the gift-size distribution exponent 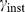.

**Figure 5:**
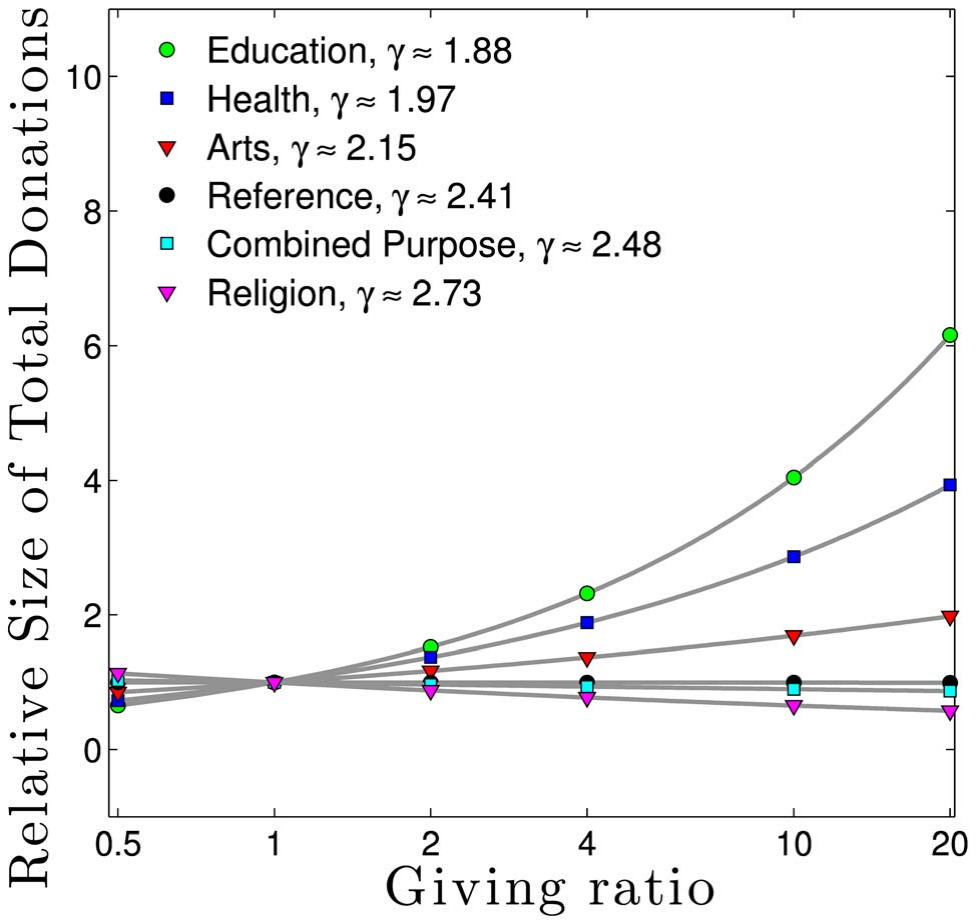
Examples of multipliers as a function of total donations and institutional categories as calculated by Equation 8. The X-axis measures the size of an individual's total donations relative to that of the index case of size 1. We calculated values of 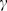 from the average of all years of data shown in Figs. 1 and 13C. Education 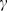 is an average of *γ*'s from University of Vermont and Albert Einstein School of Medicine; Health 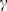 is from Mt. Sinai Hospital, Arts 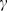 is from Flynn Theater, Combined Purpose 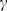 is from United Way of Chittenden County, Reference 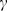 is from IRS 2001 charitable deductions Fig. 4, and Religion 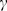 is from Fig. 13C. Our model breaks down at extreme high and extreme low incomes where the multiplier could calculate a gift that would exceed 100% of that persons total charitable giving.

Working from our data sets, we show in Fig. 5 multipliers for six types of institutions, using an income of $25,000 as an arbitrary reference for convenience.

We see that the multiplier varies strongly across income level and institutional type. Consider for example that the United Way, which has 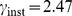, serves as our example for combined purpose funds. Because the United Ways gift-size distribution is fairly close to that of the populations giving distribution (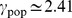), the multiplier is close to unity. Thus, if a person with a total donation level of 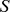 directs a certain fraction of their charitable dollars to the United Way, we expect a person with a total donation 10 times as large, 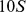, to also direct a similar fraction of their charitable dollars there as well resulting in a gift approximately 10 times larger (Fig. 5, blue squares). The multiplier here is 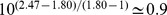, which means as a percentage of his total giving, gift from the wealthier person is 0.9 times the gift from the less wealthy person, but in absolute terms, the gift is 9 times larger because his total donation level is 10 times larger.

By contrast, for the University of Vermont for which 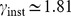, the multiplier now depends strongly on income level. If the same person with a total donation of 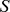 were now to direct some of their charitable dollars to the University of Vermont, the higher income person would give a multiplier of 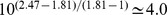, times more of their annual total donations to the same institution (Fig. 5, green circles). Note that the absolute value of the gift has increased 40 times: 10 times from the larger income, and 4 times from the multiplier effect.

**Figure 6:**
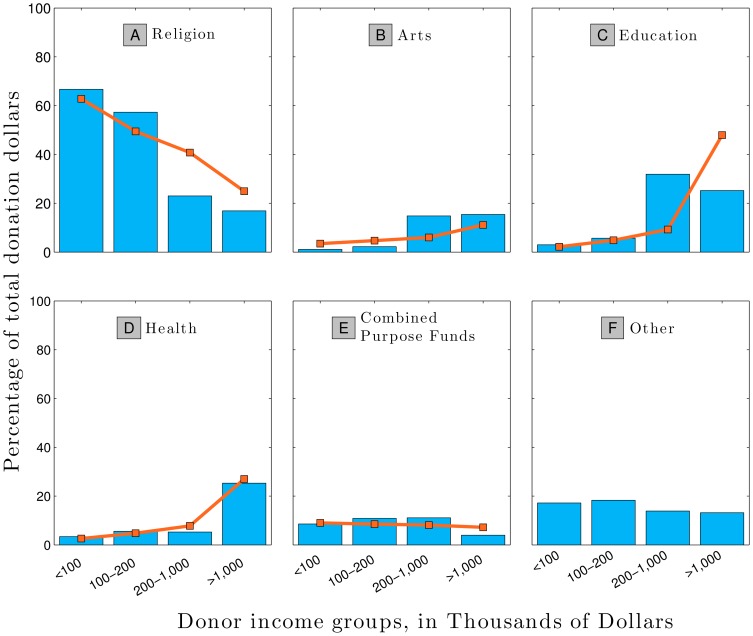
Allocation of donor giving choices as a function of income. Columns represent 2005 donor survey data [11]. Connected squares represent our model of multipliers calculated from the values of 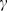 described in Fig. 5. The multiplier model agrees qualitatively with the donor survey data.

We can superimpose these multipliers onto the 2005 Study on Charitable Giving by Income Group data. We do so in Fig. 6 which is a rearrangement of the same data displayed in Fig. 2, binned according to the IRS income data in Fig. 4. The columns in Fig. 6 represent data collected from surveys of individual donors. The lines are the calculated multipliers, shown in Fig. 5. These are two independent data sets: the former represents data from the gift givers, and the latter is represents data from the gift receivers. They agree qualitatively, describing the same story but from different perspectives.

### A proposed mechanism for varying slopes

We have so far been able to describe how giving patterns must vary across institutional type as a function of institutional giving profiles and donor wealth. We now attempt to explain in part the origin of these variable donation patterns.

In many systems where multiple, dependent power-law size distributions appear, the exponents involved are typically related through simple algebraic expressions [12]. And while exponents may be tun-able as a function of independent model parameters [13], smoothly varying relationships between scaling exponents—the kind we have here–are unusual. Thus we seek to explain part of the giving mechanism that connects 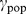 to 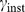 as being something more than merely “Power-Law In, Power-Law Out” (PLIPLO).

**Figure 7:**
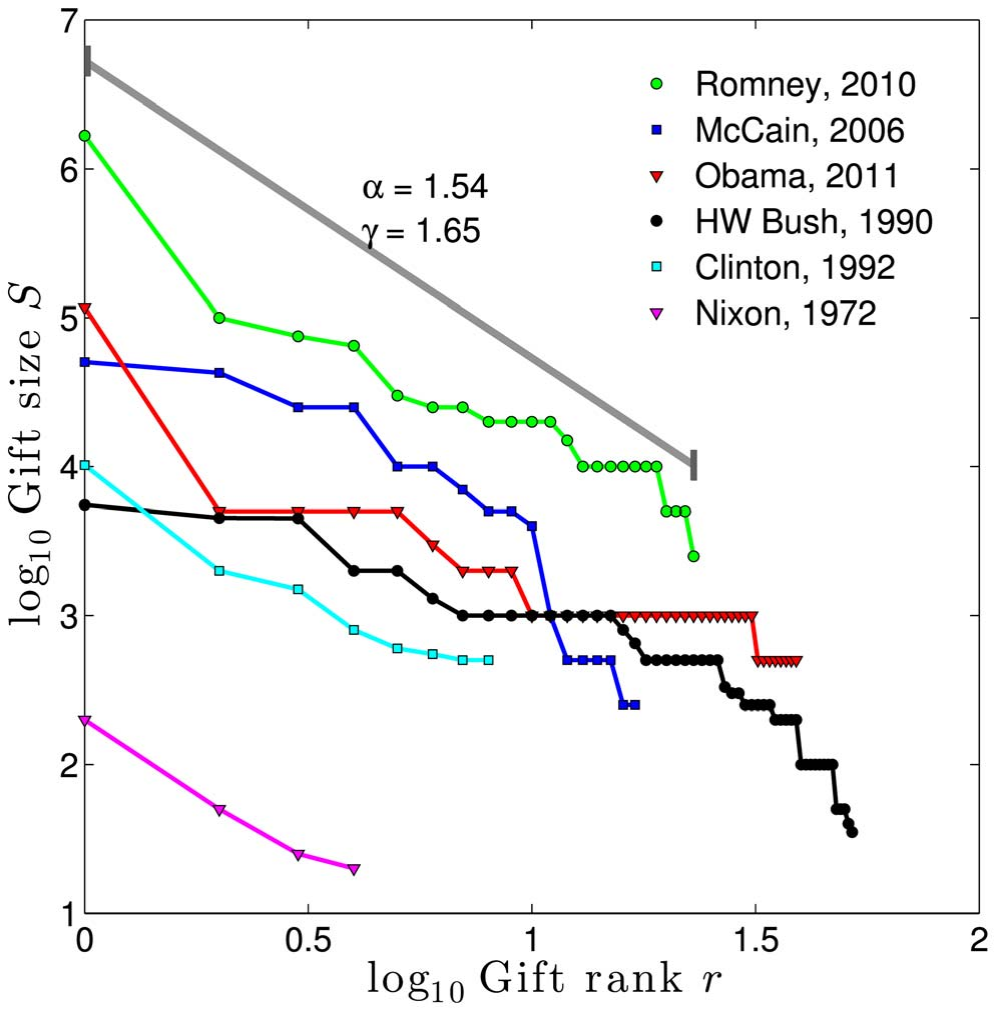
Charitable gifts of candidates for the United States President from their publicly released federal tax returns. Again due to finite size bias of maximum likelihood methods, we adopted linear regression for fitting the distribution scaling parameter 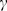. The included fit is for President Romney's gifts during the year of 2010. We include the fitted *γ*'s for each president and the range of their fit in the File S1 as Table S3, and we show comparisons to other distributions in Table S4.

We start by looking at the giving behavior of individual donors, who will differ in terms of the number of gifts they make, the size of these gifts, and their personal ranking of target institutions. They may choose to make their largest gift to health, their next largest to education, and so on. We would like examine data that characterizes the giving behaviors of these donors, such as through examination of itemized charitable deductions on federal tax returns. While this private information is generally inaccessible, some presidential candidates have released their tax returns publicly, and we can use this data as a rough guide. Fig. 7 shows itemized deductions for several candidates, plotted on log-log scales. These donations, ranked largest to smallest, are visually consistent with an approximate power law Zipf distribution with gamma ranging from 2 to 3, with an average around 2.5 (see File S1, Tabs. S3 and S4). For simplicity, we will again presume that gifts made by individual donors can be adequately described by a power law Zipf distribution. We can then propose a mechanism that uses donor choices to explain the different gammas we see among philanthropic institutions.

**Figure 8:**
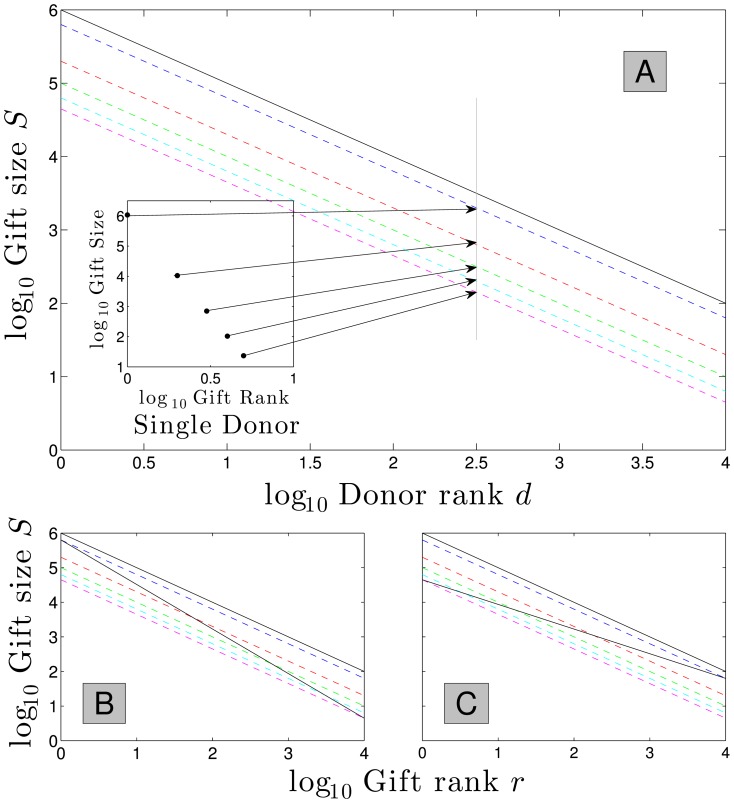
Model for differing institutional values of 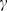. **A.** Gift distribution using 2001 IRS deduction 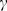 = 2.41 ranks the total of each donor's gifts (solid black line), and value of donor gifts 1 through 5 (dotted lines). Inset shows the five gifts made by donor of rank #316 (2.5 = 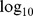
316) according to a donor 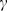 = 1.8. **B.** Institution gift distribution attracting top gift from donor 1, and 5th gift from donor 10,000, 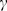 = 2.08 per Eq. (9). **C.** Institution gift distribution attracting 5th gift from donor 1, and top gift from donor 10,000, 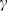 = 3.04 per Eq. (9).

In Fig. 8A, we assume a gift size distribution with 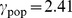 and plot the top five gifts using a rough estimate of 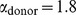. The inset shows the head of the Zipf distribution for an example donor.

Fig. 8B shows the gift distribution for an institution with strong appeal to the #1 donor garnering their top gift, but interest in this institution monotonically decreases among ranked donors until it attracts the 5th gift from the final donor. This generates a low 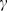 consistent with, for example, the Einstein School of Medicine illustrated in Fig. 3. Fig. 8C shows the opposite arrangement of donor appeal, leading to a high 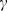 profile more typical of a religious institution.

Following this prescription we can straightforwardly derive an institution's Zipf exponent 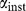 (and corresponding 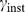) as a function of a donor's Zipf exponent 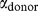, the population's Zipf exponent 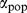 (e.g., from the IRS charitable deduction distribution), the rank of the first donor's and final donor's gift choice, and the number of donors 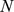, As defined by our power law model, such a relationship is linear in log space, the slope of which equals 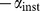. We have
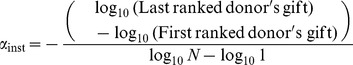
where the size of the first ranked donor's gift is given by
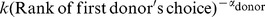
and the size of the last ranked (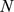 th) donor's gift is
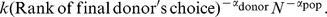
Substituting these equations into the equation for slope and simplifying gives the relationship we seek:
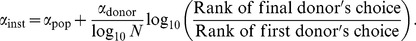
(9)For the example Fig. 8B and C, the above gives a range of 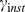 from around 2 to 3. While the rank of first and final donors' choices are fixed integers, the relationship will in practice be statistical.

## Discussion

Based on our findings, we are able to provide several recommendations and observations for fundraisers.

### The Top-12 Rule

When undertaking a capital campaign, an institution will want to estimate the fundraising capacity of its community. Capital campaigns tend be be a more focused fundraising effort targeting fewer donors than the annual campaigns reported in this paper. We have collected some preliminary data suggesting an institution's capital campaign 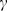 tends to be less than that of its annual campaign, resulting in a more extreme distribution of gifts. Dove reports that the top 10 to 15 donors commonly account for 50 to 70 percent of total funds raised [14]. Similarly, a professional consultant estimates capital campaign fundraising capacity using a rule-of-thumb that the top 12 donors will contribute 65% of the revenue [15]. For the sake of discussion, let us refer to this as the top-12-rule. If we can estimate an expected 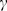 for the campaign, have an idea of the expected number of donors N, and know how much to expect from the 12 largest gifts, we can calculate a gain factor 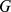 that will give an estimate for the campaign total:
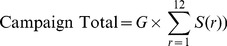
(10)where
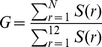
(11)


**Figure 9:**
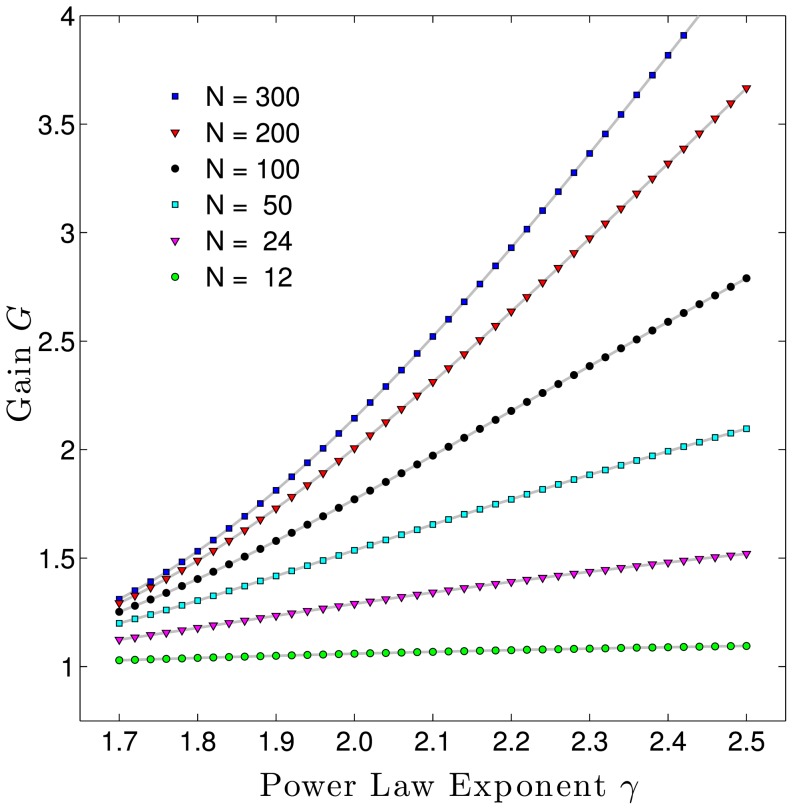
Expected total amount raised in comparison to amount raised by top 12 donors. For low 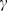 institutions, a larger number of donors has a relatively small effect on the total raised. For higher 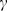 institutions, a large donor pool has a greater effect on the total raised.

When we apply a power-law model of philanthropic giving to the top-12-raises-65% rule, we can find circumstances for when this rule applies, and when it does not. Fig. 9 confirms that for campaigns with values of 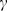 in the 1.8 to 1.9 range (e.g., for higher education), the total raised is about 1.5 times that of the top 12 donors, and increases only marginally for a donor pool total of 300 versus 100. But the rule grossly underestimates the total raised for institutions with larger values of 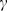. For a campaign with a 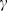 around 2.3 (e.g., for combined purpose funds) we expect the total raised by 100 donors to exceed twice that of its top 12, and that 300 donors would triple the total of its top 12. This analysis would suggest that the top-12-raises-65% rule is a poor fundraising estimator for higher values of 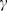 typical of combined purpose funds and religious organizations, especially for campaigns with a high number of expected donors.

Note that Fig. 9 is predicated on the assumptions that we can identify the top 12 donors for a group of a given size and that a power-law distribution of gifts applies throughout that donor pool. These assumptions no longer apply if we then increase the size of the original donor pool, because gifts from the new donors will not add serially to the tail of the distribution, but will populate all positions throughout the distribution and may exceed some of the original top 12 gifts as the pool is enlarged. This would have the effect of raising somewhat more money than predicted by Fig. 9.

In other words, as a campaign extends its original scope it may receive a gift within or greater than the original top 12. For a power-law model of growth, the total amount raised tends to grow super-linearly with the total number of donors: the *expected* amount raised from 100 donors is more than twice the *expected* amount raised from 50 donors (provided the expanded pool of donors has the same characteristic wealth distribution and interest in the organization as the original pool). This trend, however, is subject to great variability, and is more pronounced for smaller than larger values of 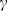. The expected largest gift from this enlarged group of donors follows the scaling:
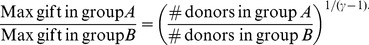
(12)


**Figure 10:**
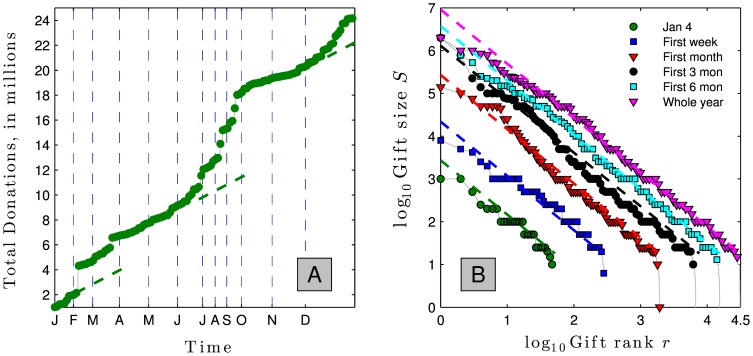
Panel A: Accumulation of gifts to University of Vermont in 2010 in the order they were received demonstrates super-linear growth. Accumulation appears linear until an uncommonly large gift is received. Panel B: Trend lines intersect the vertical axis at the expected maximum gift and show how the expected maximum gift grows with the number of donors according to Eq. (9).

The following example demonstrates the haphazard variability that this relationship is subject to. Fig. 10A shows the accumulating total in the order that gifts were received at University of Vermont in 2010. The total appears to grow linearly for a while, then jumps upwards when an exceptionally large gift is received.

Fig. 10B shows these gifts broken down into various time frames. The dotted lines demonstrate how the *expected* largest gift grows (per Eq. (9)) as the number of donors increases. Not surprisingly, the *actual* largest gift shows substantial variability around this predicted value.

### The 80-20 Principle and the Fundraising Pyramid

**Figure 11:**
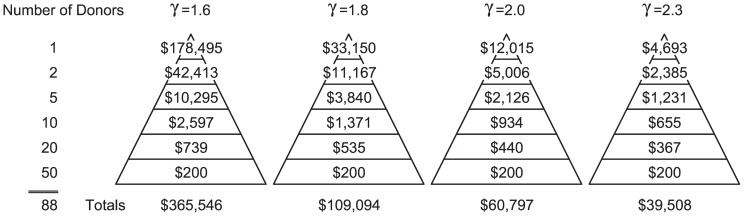
Fundraising pyramids customized to an institutions 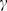. Using a power-law model of fundraising, low 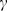 institutions should plan for and request much higher top level gifts than high 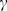 institutions.

Commonly the gifts table (fundraising pyramid) is predicated on Vilfredo Pareto's 80/20 principle; that 80% of funds are raised from 20% of donors [6]. Pareto originally founded his principle on his observation of the power-law size distribution of wealth in Italy [16] (the 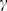 for an 80/20 fundraising relationship varies based on the number of donors, ranging from 1.82 for 100 donors, to 2.04 for 5,000 donors). By knowing an organization's 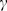 and donor pool size, a fundraiser can develop a giving pyramid specific to that organization's gift distribution, rather than using a generic 80/20 pyramid. Fig. 11 shows four pyramids, each calculated for an organization with a specific 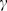. For each pyramid the lowest level of donations is set at $200. Organizations with lower values of 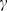 should plan for much larger gifts at the higher levels than organizations with higher values of 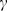, and should expect to raise much more from the same number of donors.

### What 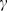 says about an organization's fundraising capacity and robustness

**Figure 12:**
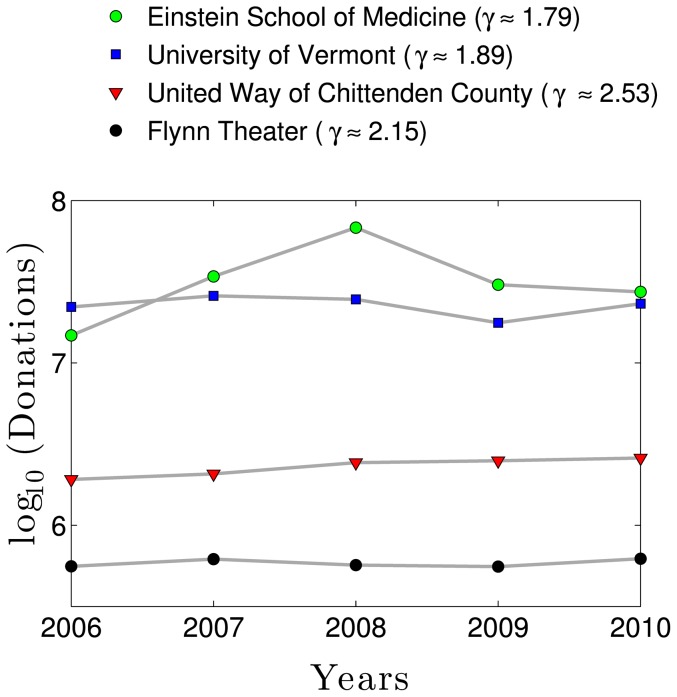
Low 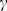 organizations can expect to have greater year-to-year fundraising volatility than higher 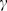 organizations. The 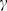 values reported here are averages for all of the years shown in Fig. 1.

Low values of 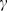 generally describe organizations whose largest gifts are extremely large in proportion to the total amount raised. For example, for a campaign with a 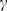 of 1.81 (e.g., higher education) and 1000 donations, the lead gift would be expected to be about 30% of the total raised. But for a campaign with a 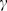 of 2.47 (e.g., combined purpose fund) the lead gift would be expected to be about 5% of the total. In contrast to the predictability of mid-level gifts, lead gifts are subject to enormous variability about their expected value, as can be seen by their divergence from the projected line of slope in most of the data examples presented in this paper. This means that while low 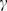 institutions are more likely to enjoy the benefit of extremely large gifts, their annual fundraising total is highly dependent on the gifts of those top few donors and becomes subject to significant year-to-year variability (Fig. 12). In contrast, high 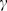 institutions are likely to experience more stable year-to-year totals. Note that United Way of Chittenden County and Einstein School of Medicine have a similar sized donor base, demonstrating the fundraising power of a low 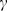.

### Misleading Effect of Multiple Donations per Donor

**Figure 13:**
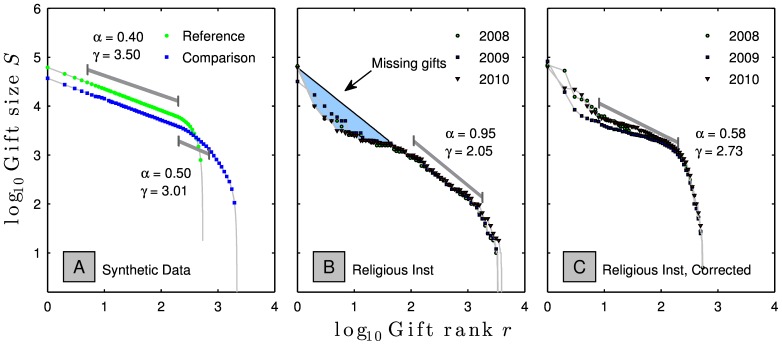
**A.** ‘Reference’ synthetic power law giving distribution with a largest gift of $62,000, 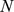 = 542 total donors of which the largest 200 follow a power law with 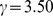 is created and labelled Reference, with a fit shown between the 5th and 200th gifts. A comparison is created where each gift from the reference distribution is split into 60%, 25%, 10%, and 5%. A false shoulder is created with a 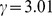, fitting the slope between the 200-th and 700-th donors. **B.** Gift-size data from an anonymous religious institution. The blue region appears to represent around $100,000 of unrealized potential in the $1,000 and above range. The power law slope of 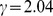 is found by fitting the gifts from donor 110 to donor 1800, over the region that appears flattest. **C.** The giving data from panel **B** corrected for multiple donations, where now 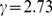 is found between the 8th and 200-th donors.

For proper analysis, data for a given time period must reflect a single total of gifts from each donor. If the donor has made multiple gifts, and these gifts are recorded separately, the number of donors, 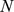, will be falsely inflated. As shown in Fig. 13A, this can create a false and misleading shoulder on the institution's Zipf plot and lead to a miscalculation of 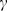. Fig. 13B appears to show gifts from 3,500 donors to an anonymous religious institution, with 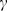 of 2.04. There appears to be about $100,000 of unrealized potential from the larger gifts. In fact, there were only 500 donors, but many of them had made multiple smaller gifts throughout the year. In panel C, for each year we properly summed multiple gifts by single donors into a single total for each donor, and see that 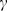 of 2.04 in Panel B was entirely due to a false shoulder effect. The correctly measured 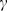 of 2.73 is more consistent with that predicted from the donor survey data for religious institutions from Fig. 6. We now see that the largest gifts in fact exceeded expectations.

## Conclusions

The distribution of gifts received by nonprofit institutions is approximately consistent with a power-law size model. Individual institutions, and possibly broad of categories institutions, have their own characteristic scaling exponent 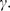 Fundraising projections modeled on power laws may be useful for predicting the success of a given campaign, and for affecting the strategic planning of a campaign.

Future study should assemble a larger database to see if our findings are consistent, to study if there is a predictable relationship between the values of 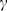 for an institution's annual fund and its capital campaigns, and to capture values of 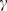 for new philanthropic categories such as human services and the environment. Different regions across the globe and within countries may show characteristic local variations for values of 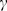 for income, overall giving, and by category of institution. Gifts from private individuals account for 73% of giving; family foundations, corporate giving, and bequests account for the remainder [1]. Analysis of these disparate funding sources may find characteristic values of 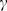 for gifts based on the category of their source in addition to the category of their destination.

## Methods

All data sets can be downloaded from our present paper's online appendix which is located here: http://www.uvm.edu/storylab/share/papers/gottesman2014a/.

In this section, we provide some salient details regarding our data sets, and we describe fitting gift size distributions to a number of potential candidate forms, employing methods of Maximum Likelihood (ML) for the estimation of parameters [8]. Unsurprisingly, a pure power law decay does not fit the data with great precision. Nevertheless, we justify our use of a power law size distribution as a reasonable, if rough, characterization of philanthropic gift size distributions–very much in the manner of standard linear regression–and hence a suitable building block for our analyses. Larger, much more exhaustive data sets across all kinds of institutions will be required to strongly advance our knowledge of philanthropy beyond what we have achieved here.

### Details of philanthropic data

For all sources of data in this present work, we made no distinctions as to whether the donor of a gift was a living person, a bequest, a foundation, or a corporation. In the 5 year period 2006 through 2010, the sources of total giving in dollars were divided as 73% individuals, 8% bequests, 14% foundations, and 5% corporate [1].

For the institutions we analyse, gift size specifics were as follows:

- Albert Einstein School of Medicine, University of Vermont, ECHO Aquarium and Science Center, and the Flynn Theater provided data for all gifts received over 5 years. Multiple gifts by a single donor over a single year were not identified by these institutions, and hence not summed into a single gift.
- Mount Sinai Hospital reported all gifts and was able to identify multiple gifts per year from individual donors; these were summed into a single gift per donor per year.
- United Way of Chittenden County likewise identified multiple gifts which were summed into a single gift per donor per year, but was able to provide data only on gifts received individually. Some workplaces collect United Way donations and then send a lump sum: these sums do not reflect individual gifts and were not reported to us.
- The anonymous religious institution described their gifts both as multiple donations per donor per year and a summed donation per donor per year, permitting construction of Fig. 13.

Individual donations from United States Presidents and candidates were obtained directly from their tax returns for the stated years. This data is available directly at http://www.taxhistory.org/www/website.nsf/Web/PresidentialTaxReturns. In addition, we include the data presented here in a CSV file.

### Scaling parameter fitting

In general, we use the ML method to fit our scaling parameter 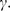 However, for small data (e.g., presidential gifts and limited tax data) the ML method is biased from the finite size and we use a linear regression for a rough estimate. To determine the portion of our data that is best power-law behaved, the minimization of the Kolmogorov-Smirnoff statistic 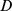 proved to be inconsistent across our data due the multiple minima of the statistic 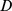 (see Fig. S1). For this reason, we empirically chose the scaling regions (i.e., the cut offs).

In Tab. S1 (File S1), we report results for fitting power-law decay distributions using the ML approach, and in Tab. S3 (File S1) we perform the same analysis for presidential gifts. We used code from both Clauset and Alstott, as well as our own [8], [9]. We note that as argued by Alstott et al., the 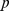-value of the fitted distribution becomes less useful for large data sets because the Monte Carlo generated distributions become nearly perfect [9]. Since our data is large, we find that in general none of the synthetically generated data sets has 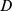 greater than for the real data (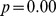), but this does not rule out power law behavior.

We then turn to the comparison to other distributions in Tab. S2 in File S1 (and Tab. S4 in File S1 for presidential gifts), and find that the power law is at least reasonably supported in most cases. The only test for which statistical significance was at least as common is for the exponential distribution, and in all cases the power law was favored. For all distributions where the cutoff power law test was significant, in particular the Einstein School of Medicine, we find that the cutoff power law is favored, and would be the most likely distribution.

## Supporting Information

Figure S1
**The Kolmogorov-Smirnoff statistic** 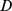 **plotted over** 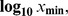 **where** 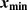 **is the minimum value fit for power law behavior, for the United Way of Chittenden County over the years 2006–2010.** 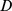 is generated from the ML estimate. Existence of multiple minima in our data indicate that there are multiple possible fitting regions for which the KS statistic suggests a good fit. The variability of this value over each year plotted produced widely varying scaling parameters 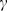, and thus could not be safely used.(TIFF)

File S1 **Supporting tables S1–S4.**
(PDF)

Dataset S1 (ZIP)
