# Supplementary material for: Collective Philanthropy: Describing and Modeling the Ecology of Giving
Source: PLoS One. 2014 Jul 1;9(7):e98876. doi: 10.1371/journal.pone.0098876 (PMC4077655; doi:10.1371/journal.pone.0098876)
Supplement: File S1 — Supporting tables S1–S4. (PDF) [file pone.0098876.s002.pdf]

Supplementary material

Supplementary Tables with Legends

**Institution**	**Year**	$\langle \mathbf{x} \rangle$	σ	$\mathbf{x}_{\max}$	γ	Range	**D**	**p**
Mount Sinai Hospital	2009	17618.40	450408.65	37259947	1.92 ± 0.08	1 to 90	0.12	0.00
	2010	19348.18	429587.88	27885708	2.02 ± 0.10	1 to 90	0.10	0.00
Einstein School of Medicine	2006	3247.30	46940.29	2000000	1.79 ± 0.02	1 to 2000	0.11	0.00
	2007	4768.09	78762.48	5350000	1.71 ± 0.01	1 to 2000	0.15	0.00
	2008	10385.80	199751.68	10200000	1.80 ± 0.01	1 to 2000	0.21	0.00
	2009	5212.92	139468.89	10000000	1.84 ± 0.01	1 to 2000	0.15	0.00
	2010	4917.94	61893.49	2000000	1.80 ± 0.06	1 to 2000	0.15	0.00
Univeristy of Vermont	1974	155.76	2811.94	200000	1.94 ± 0.01	3 to 794	0.18	0.00
	1980	284.31	5284.36	326000	1.85 ± 0.03	3 to 794	0.11	0.00
	1990	350.23	5382.45	500000	2.16 ± 0.01	3 to 794	0.38	0.00
	2000	805.33	15120.53	1488000	1.71 ± 0.03	3 to 794	0.09	0.00
	2010	741.40	17029.10	2000000	1.81 ± 0.05	3 to 794	0.13	0.00
United Way, Chittendon County	2004	441.71	1133.02	30000	2.77 ± 0.04	1 to 316	0.21	0.00
	2005	464.47	1444.26	50000	2.58 ± 0.22	1 to 316	0.13	0.00
	2006	456.86	1199.92	25000	2.42 ± 0.05	1 to 316	0.07	0.00
	2007	456.16	1279.14	30000	2.42 ± 0.14	1 to 316	0.07	0.00
	2008	287.53	1089.92	45460	2.53 ± 0.00	1 to 316	0.14	0.00
	2009	278.93	1122.44	56500	2.55 ± 0.08	1 to 316	0.12	0.00
	2010	287.58	1271.10	70518	2.47 ± 0.09	1 to 316	0.08	0.00
ECHO Science Museum	2005	977.77	3153.41	25000	1.66 ± 0.03	2 to 88	0.20	0.00
	2006	951.16	3415.22	25000	1.59 ± 0.02	2 to 88	0.28	0.00
	2007	941.61	3161.08	25000	1.59 ± 0.07	2 to 88	0.31	0.00
	2008	956.88	2688.31	20000	1.56 ± 0.01	2 to 88	0.26	0.00
	2009	676.84	2098.96	20000	1.73 ± 0.15	2 to 88	0.17	0.00
Flynn Theater	2006	241.87	1528.82	65065	2.18 ± 0.04	1 to 2000	0.26	0.00
	2007	268.54	1732.33	60000	2.15 ± 0.05	1 to 2000	0.25	0.00
	2008	248.00	1015.39	27500	2.15 ± 0.00	1 to 2000	0.22	0.00
	2009	242.90	1212.42	40000	2.18 ± 0.04	1 to 2000	0.23	0.00
	2010	246.13	1606.43	70000	2.09 ± 0.05	1 to 2000	0.22	0.00

Table S1. Summary statistics of all of the donation data is presented. The reported γ and range are fit with the MLE method, and the $x_{\min}$ which was found to minimize the Kolmogorov-Smirnoff statistc **D** is reported along with **D** itself. In this case, lower values of **D** indicate a better fit.

Institution	Year	p	Log-Normal LR	Log-Normal p	Exponential LR	Exponential p	Stretched Exp. LR	Stretched Exp. p	Cutoff Power Law LR	Cutoff Power Law p
Mount Sinai Hospital	2009	0.00	-0.21	0.67	31.80	**0.01**	-0.19	0.82	-0.53	0.30
	2010	0.00	-0.00	0.99	47.31	**0.00**	0.46	0.60	-0.23	0.50
Einstein School of Medicine	2006	0.00	-6.22	**0.03**	378.82	**0.00**	-7.06	**0.03**	-8.31	**0.00**
	2007	0.00	-0.30	0.59	17.65	**0.01**	-0.35	0.61	-0.67	0.25
	2008	0.00	-1.03	0.37	1235.22	**0.00**	0.71	0.81	-2.85	**0.02**
	2009	0.00	-2.48	0.13	578.27	**0.00**	-2.75	0.22	-5.82	**0.00**
	2010	0.00	-1.52	0.22	842.87	**0.00**	-0.64	0.80	-5.19	**0.00**
Univeristy of Vermont	1974	0.00	-0.39	0.54	20.93	**0.00**	-0.49	0.54	-1.17	0.13
	1980	0.00	-0.72	0.41	82.27	**0.00**	-0.81	0.47	-1.82	**0.06**
	1990	0.00	-0.94	0.36	23.05	**0.01**	-1.11	0.34	-1.79	**0.06**
	2000	0.00	-0.65	0.45	30.59	**0.00**	-0.78	0.44	-1.52	**0.08**
	2010	0.00	$-\infty$	**0.00**	7.75	**0.02**	0.39	0.34	-0.00	0.94
United Way, Chittendon County	2004	0.00	-0.46	0.47	28.75	**0.00**	-0.53	0.55	-1.29	0.11
	2005	0.00	-0.08	0.77	54.69	**0.00**	0.36	0.74	-0.69	0.24
	2006	0.00	-0.12	0.71	68.71	**0.00**	0.44	0.71	-0.85	0.19
	2007	0.00	-0.61	0.43	48.21	**0.00**	-0.65	0.57	-1.64	**0.07**
	2008	0.00	-0.13	0.72	46.52	**0.00**	0.14	0.90	-0.71	0.23
	2009	0.00	-0.35	0.55	48.39	**0.00**	-0.28	0.80	-1.15	0.13
	2010	0.00	-0.32	0.58	35.25	**0.00**	-0.30	0.77	-0.90	0.18
ECHO Science Museum	2005	0.00	-2.47	0.25	31.43	**0.04**	-3.04	0.21	-3.56	**0.01**
	2006	0.00	-0.20	0.69	1.42	0.57	-0.28	0.68	-0.53	0.30
	2007	0.00	$-\infty$	**0.00**	4.56	**0.03**	0.20	0.35	0.00	1.00
	2008	0.00	$-\infty$	**0.00**	4.28	**0.00**	0.29	0.19	0.00	1.00
	2009	0.00	-0.87	0.47	31.48	**0.01**	-1.23	0.44	-2.51	**0.03**
Flynn Theater	2006	0.00	-0.52	0.46	272.93	**0.00**	0.32	0.87	-2.80	**0.02**
	2007	0.00	-0.06	0.80	4.53	0.14	-0.08	0.86	-0.26	0.47
	2008	0.00	-0.56	0.45	303.73	**0.00**	0.38	0.86	-3.35	**0.01**
	2009	0.00	-0.25	0.63	281.34	**0.00**	1.11	0.59	-2.16	**0.04**
	2010	0.00	-3.96	**0.07**	129.19	**0.00**	-4.61	**0.06**	-6.78	**0.00**

Table S2. The results of the Likelihood-Ratio and its associated p-value are reported for different distributions. Here, positive values lend support to the Power Law and negative values to the other stated distribution. The significance of the **LR** is **p**, where low values of **p** indicate a trustworthy **LR**. Values for which $\mathbf{p} < 0.05$ are bolded.

**Name**	**Year**	$\langle \mathbf{x} \rangle$	σ	$\mathbf{x}_{\max}$	γ_{MLE}	$\mathbf{x}_{\min}$	**D**	**p**	γ_{LS}	**Range**
Romney	2010	94456.52	336755.59	1670000	2.09 ± 0.25	10000	0.11	0.00	1.65 ± 0.13	1 to 23
McCain	2006	11037.59	15077.01	50500	1.88 ± 0.28	4000	0.20	0.00	1.47 ± 0.12	1 to 17
Obama	2011	4413.59	18330.81	117130	3.16 ± 0.39	1000	0.13	0.00	1.96 ± 0.26	1 to 39
HW Bush	1990	749.94	1116.48	5521	3.35 ± 0.61	1000	0.17	0.00	1.79 ± 0.19	1 to 52
Clinton	1992	2083.75	3117.79	10220	2.06 ± 0.43	550	0.15	0.50	1.69 ± 0.12	1 to 8
Nixon	1972	73.75	73.77	200	2.16 ± 0.58	20	0.18	1.00	1.58 ± 0.11	1 to 4

Table S3. Summary statistics of all of the Presidential donation data.

Name	Year	p	Log-Normal LR	p	Exponential LR	p	Stretched Exp. LR	p	Cutoff Power Law LR	p
Romney	2010	0.00	$-\infty$	**0.00**	28.63	**0.00**	0.70	0.15	0.00	1.00
McCain	2006	0.00	-0.39	0.60	0.10	0.96	-0.56	0.58	-0.75	0.22
Obama	2011	0.00	$-\infty$	**0.00**	55.53	**0.00**	2.67	**0.01**	0.00	1.00
HW Bush	1990	0.00	$-\infty$	**0.00**	5.27	**0.01**	0.55	**0.09**	0.00	1.00
Clinton	1992	0.50	$-\infty$	**0.00**	2.58	0.15	0.06	0.78	-0.02	0.83
Nixon	1972	1.00	$-\infty$	**0.00**	1.11	0.41	0.03	0.85	-0.03	0.82

Table S4. The results of the Likelihood-Ratio and its associated p-value are reported for the presidential candidates.
